# Data for the synthesis of pyrogallol-formaldehyde aerogels using two acid catalysts oxalic acid y hydrochloric acid

**DOI:** 10.1016/j.dib.2019.103866

**Published:** 2019-03-19

**Authors:** Jhonatan R. Guarín Romero, Marlon Bastidas-Barranco, Paola Rodríguez-Estupiñán, Liliana Giraldo, Juan Carlos Moreno-Piraján

**Affiliations:** a Facultad de Ciencias, Departamento de Química, Grupo de Investigación en Sólidos Porosos y Calorimetría, Universidad de los Andes, Carrera 1ra # 18A-12, Bogotá, Colombia; b Facultad de Ingeniería, Grupo DestaCar, Universidad de la Guajira, Km 5 Via Maicao, Riohacha, Guajira, Colombia; c Departamento de Química, Facultad de Ciencias, Universidad Nacional de Colombia, Carrera 30 # 45 - 03, Bogotá, Colombia

**Keywords:** Aerogels, Pyrogallol-formaldehyde, Oxalic acid, Hydrochloric acid

## Abstract

Aerogels are extremely porous materials with large pore volumes and low bulk densities. Their unique structure imparts extraordinary properties and wide applications. The synthesis of pyrogallol-formaldehyde xerogels has been reported using HClO_4_ as a catalyst, but according to the literature review the synthesis of aerogels of these materials has not been documented. In the present work, the data for the synthesis of aerogels pyrogallol-formaldehyde are presented using oxalic acid and hydrochloric acid as catalysts. Also includes the data of the characterization of these materials by Infrared spectroscopy, thermogravimetric analysis Tg-DTG, Physisorption of N^2^, Raman Spectroscopy, X-ray Diffraction (XRD) and Scanning Electron Microscopy (SEM). It was determined that the use of these precursors of the synthesis of aerogels in acid medium, leads to the obtaining of microporous solids with a high value of the surface area, the material with the highest value of this parameter has been CAePF OA550 at have a BET area value of 1066 m^2^ g^−1^.

**Table:** Specifications table

Subject area	*Chemistry*
More specific subject area	*Materials science: Carbons aerogels*
Type of data	*Tables, images, text, graphs and figures*
How data was acquired	*Infrared Spectroscopy Shimazu (IRT racer-100, Columbia, SC, USA), TGA–DTA (Hitachi model*7200*), Nitrogen isotherm of N*_*2*_*at −196* °*C (IQ2, Quantachrome Inc.), Raman Spectroscopy (HORIBA Scientific instrument (Newark, NJ, USA), XRD (Rigaku RU-300), SEM (JEOL JSM* 6490-LV *microscope (Peabody, MA, USA))*
Data format	*Raw and analysed.*
Experimental factors	*The aerogels were prepared by the sol-gel method performing a supercritical drying with CO*_*2*_*, using two acid catalysts, one weak and one strong oxalic acid and hydrochloric acid respectively, the P/C ratio of 25, 500 and*1000*was varied to obtain 6 samples total.*
Experimental features	*The six samples of the organic aerogels were characterized by: IR for this a small amount of the organic aerogel was mixed with KBr, by Tg-DTG the loss of mass and the stages occurring during the carbonization process was determined, N*_*2*_*physisorption, for this they were degassed at 250 °C and a vacuum of 1* × *10*^*−5*^*mbar for a period of 3 h, for the Raman measurements no additional preparation of the samples was carried out. Samples, this analysis was performed in a range of 500 -*2500*cm*^*−1*^*using a laser of* 532 nm *and a target x10 NIR. For the SEM analysis the samples were degassed and coated with Au.*
Data source location	*Bogotá, Colombia, Universidad de los Andes (4° 37′ 27.6060″ N and 74° 3′ 49.1184″ W.)*
Data accessibility	*Data are provided in this article*
Related research article	*W. Djeridi, N.B. Mansour, A. Ouederni, P. Llewellyn, L. El Mir, Study of methane and carbon dioxide adsorption capacity by synthetic nanoporous carbon based on pyrogallol-formaldehyde, international journal of hydrogen energy, 42 (*2017*) 8905–8913.*

**Table:** 

**Value of the data** • In Many articles of aerogels, Resorcinol-formaldehyde materials are used as starting precursors in this work the resorcinol was changed by pyrogallol using the mixture of pyrogallol-formaldehyde. • The synthesis method is similar to the one used to obtain resorcinol-formaldehyde aerogels; however, when using other precursors, the P/F ratio has been modified. • The acid catalysis route to obtain aerogels is less used compared to the basic route, in this work this methodology has been used with two acid catalysts such as oxalic acid and hydrochloric acid. • The data obtained from the different characterization techniques will help to guide the researchers towards the possible applications that these solids may have.

## 1 Data

**Fig. 1:**
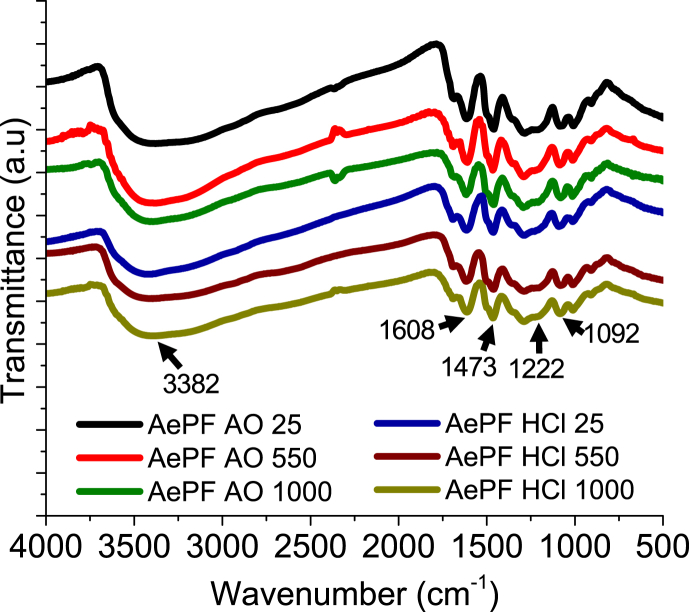
Infrared spectroscopy (IR) spectra of dry aerogels.

The Surface chemical composition of PF aerogels was analyzed with IR. The IR spectrum of a PF aerogel (Fig. 1) shows the same features to those shown by Resorcinol-formaldehyde aerogels [1], with bands at 1473 cm^−1^ associated with the CH_2_ stretching and bending vibrations, whereas the broadband at 3382 cm^−1^ includes the aromatic OH groups of resorcinol. The band at 1608 cm^−1^ comes from aromatic ring stretches, whereas medium to weak absorption bands at 1222 and 1092 cm^−1^ indicate that methylene ether linkages between resorcinol rings are present but not dominant [1].

**Fig. 2:**
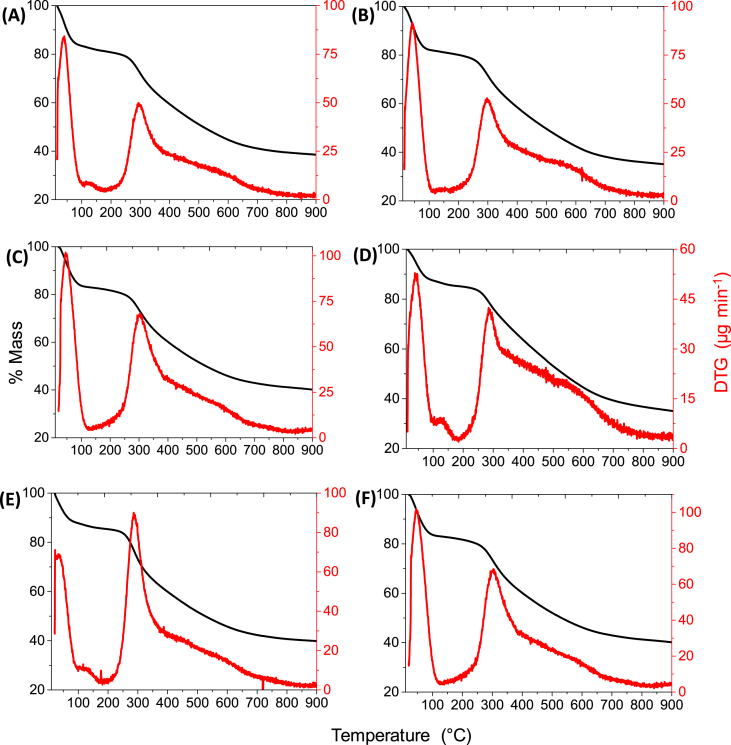
Thermogravimetric analysis of pyrogallol-formaldehyde aerogels (A) CAePFOA 25, (B) CAePFOA 550, (C) CAePFOA 1000, (D) CAePFHCl 25, (E) CAePFHCl 550, (F) CAePFHCl 1000.

**Fig. 3:**
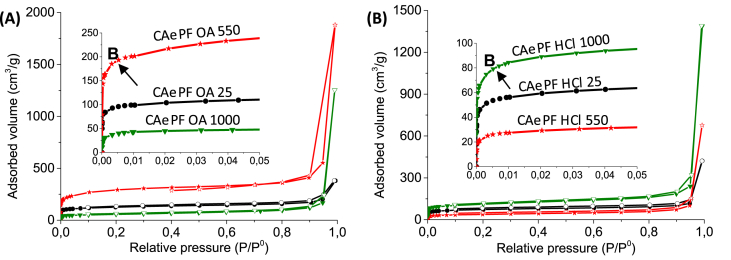
Physisorption isotherms of N_2_ at −196 °C of the carbon aerogels obtained in acidic medium (A) oxalic acid y (B) HCl.

Fig. 2 shows the weight loss versus temperature for a dried pyrogallol formaldehyde gel. The first peak appears at a low temperature (<100 °C), whereas the other peak appear at 310 °C. The first peak probably corresponds to the extraction of the remaining solvent and/or the elimination of H_2_O formed from the condensation of OH groups, while the second peak is attributed to hydrogen and oxygen atoms in the polymer network being released as CO_2_ and CH_4_ or other organic molecules, and probably to the desorption of adsorbed organic compounds [2].

(Fig. 3 shows the physisorption isotherms of N_2_, whose point B is well defined, which means that at low pressures the coverage of the monolayer has been completed and that the solids have intra-particulate pores distributed homogeneously and with size <1nm. The isotherms are type II according to the IUPAC 2015 [3] classification, therefore, there is also the presence of interparticle macrocomposites, in which multilayer adsorption was carried out at a relative pressure value close to 1 (see Table 1).

**Table 1:** BET surface areas of the samples obtained.

*Sample*	*S*_*BET*_*(m*^*2*^*g*^*−1*^*)*
CAePF OA25	465
CAePF OA550	1066
CAePF OA1000	202
CAePF HCl25	269
CAePF HCl550	137
CAePF HCl1000	404

**Fig. 4:**
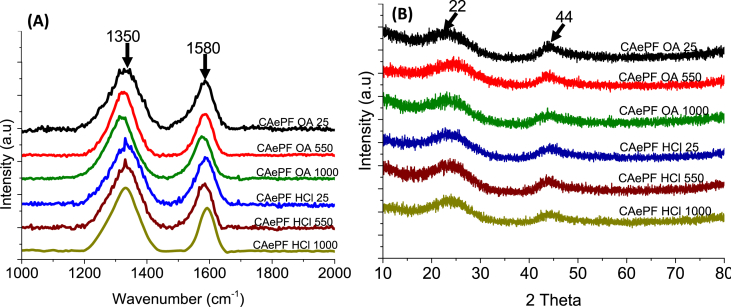
Spectrum (A) Raman and (B) DRX of aerogels.

Raman spectroscopy (Fig. 4 (A)) show two bands, the first was observed at 1350 cm^−1^, named as ‘disorder-induced’ or D1 mode, is commonly ascribed to the lack of a long range translation symmetry in disordered carbons in the graphitic layers [4]. The G band appearing at 1580 cm^−1^ is ascribed to a Raman-allowed E_2g_ resulting from ‘in plane’ displacement of the carbons strongly coupled in the hexagonal sheets [5], it is characteristic of sp^2^—hybridized C–C bonds in a two-dimensional hexagonal lattice, [4].

These data were confirmed by XRD patterns of aerogels samples show two broadened bands located at 22° and 44° (2Θ) which are respectively indexed as the reflections of a graphite-based structure (Fig. 4 (B)), the enhanced broadening of the reflections is ascribable to the structural disordering existing in carbon materials [6].

**Fig. 5:**
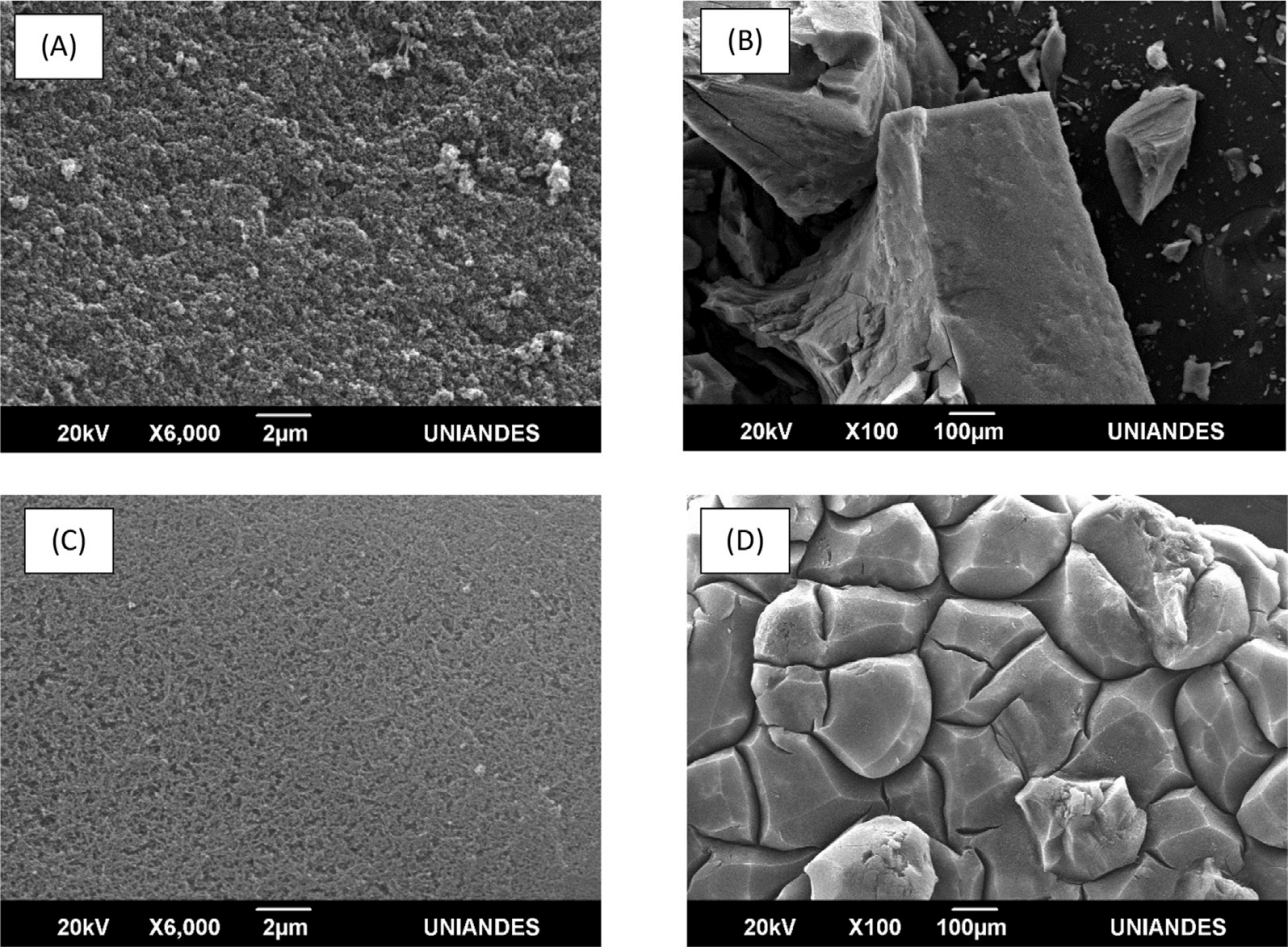
SEM de los aerogeles (A) y (B) CAePF OA 550, (C) y (D) CAePF HCl 1000.

Fig. 5 shows the SEM micrographs of pyrogallol-formaldehyde aerogels that have the best textural properties (CAePF OA 550 and CAePF HCl 1000). These images show that our samples are very porous and are characterized by nanopores. Such as we have obtained in the study of Raman and XRD, these PF composites are amorphous [7].

## 2 Experimental design, materials, and methods

### 2.1 Synthesis

Aerogels were synthesized by the sol-gel method [8]. In this process, the starting precursors pyrogallol and formaldehyde were mixed with a pyrogallol/formaldehyde (P/F) ratio of 0.33, and deionized water was added as a solvent in a pyrogallol/water (P/H_2_O) ratio of 0.0504. The catalysts used were weak and strong acid, oxalic acid and hydrochloric acid respectively, in a 25, 550 and 100 pyrogallol/catalyst (R/C) ratio. The resulting solutions were agitated for 30 min and placed in polypropylene molds. Then, the thermal treatment stage was carried out, during which hydrogels were left at 25 °C for one day, 50 °C for two days, and finally 80 °C for three days. After the hydrogels were cooled to room temperature, the solvent was allowed to exchange with acetone for three days. For supercritical drying with CO_2_, a high-pressure reactor was used at 40 °C with a pressure of 120 bar.

## 3 Structural characterization

### 3.1 Infrared spectroscopy

Fourier Transform Infrared Spectra (FTIR) of the organic aerogels were obtained in a Shimazu (IRT racer-100, Columbia, SC, USA). The pressed granules were prepared by grinding the aerogels and mixing them with KBr in an agate mortar. The spectral data were recorded at wavenumber values of 4000–500 cm^−1^.

### 3.2 Thermogravimetric analysis TG-DTG

This analysis was performed on a HITACHI STA7200 equipment. 10 mg of the organic aerogels were weighed, subjected to a nitrogen flow of 100 mL/min and carbonized with a linear heating rate of 5 °C/min up to a final temperature of 900 °C.

### 3.3 Physisorption of N_2_ at −196 °C

A sample of 0.1000 g of the synthesized carbon aerogels was degassed at 250 °C and a vacuum of 1 × 10^−5^ mbar for a period of 3 h was used to remove all the adsorbed species that could intervene in the adsorption processes (automatic IQ2 sortometer, Quantachrome Inc., Boynton Beach, FL, USA). The corresponding N_2_ adsorption isotherms at −196 °C were obtained in the above equipment with a relative pressure range between 4 × 10^−5^ and 1. The specific surface area was evaluated from the Brunauer-Emmet-Teller (BET) method [9] with the data obtained for N_2_ relative pressures (P/P^0^) in a range that meets the requirements for micropore materials (IUPAC 2015) [3].

### 3.4 Raman Spectroscopy

Raman Spectroscopy were taken on a HORIBA Scientific instrument (Newark, NJ, USA) in a range of 500–2500 cm^−1^ using a 532 nm laser and a 10 × (Near Infrared) (NIR) target. For this analysis, no additional preparation of the samples was performed. This technique was used to demonstrate that the carbonized samples had a disordered structure composed of layers of graphene.

### 3.5 X-ray diffraction (XRD)

The diffractograms of the solids were taken in a Rigaku RU-300 equipment with CuKα lamp [λ = 1.5418 Å] operated at 40 kV and 80 mA with a sweep at angles 2Θ between 10 and 80°. For this analysis the samples were sprayed in accordance with the equipment and the cell used.

### 3.6 Scanning Electron Microscopy (SEM)

This analysis was performed on a JEOL JSM 6490-LV microscope (Peabody, MA, USA). For this, a certain quantity of carbon aerogel was triturated. Before carrying out this analysis, the aerogel surface was coated with gold using the sputtering method to obtain a conductive surface. This analysis was carried out to observe the material surface.
