# Supplementary material for: Data for the synthesis of pyrogallol-formaldehyde aerogels using two acid catalysts oxalic acid y hydrochloric acid
Source: Data Brief. 2019 Mar 19;23:103866. doi: 10.1016/j.dib.2019.103866 (PMC6660616; doi:10.1016/j.dib.2019.103866)
Supplement: Multimedia Component 1 [file mmc1.doc]

Conflict of Interest Form

The authors of this investigation declare that they have no conflicts of interest in this investigation.

For the signature the author of correspondence signs on behalf of all.

Sincerely yours

Prof. Dr. Juan carlos Moreno-Piraján

Ful Professor

Universidad de los Andes

Colombia
